# Xanthohumol increases death receptor 5 expression and enhances apoptosis with the TNF-related apoptosis-inducing ligand in neuroblastoma cell lines

**DOI:** 10.1371/journal.pone.0213776

**Published:** 2019-03-14

**Authors:** Samuel Engelsgjerd, Selvi Kunnimalaiyaan, Emad Kandil, T. Clark Gamblin, Muthusamy Kunnimalaiyaan

**Affiliations:** 1 Division of Surgical Oncology, Department of Surgery, Medical College of Wisconsin, Milwaukee, WI, United States of America; 2 Department of Surgery, Tulane University School of Medicine, New Orleans, LA, United States of America; Institute of Biochemistry and Biotechnology, TAIWAN

## Abstract

High-risk neuroblastoma (NB) is lethal childhood cancer. Published data including ours have reported the anti-proliferative effect of Xanthohumol (XN), a prenylated chalcone, in various cancer types suggesting that XN could be a useful small molecule compound against cancer. The TNF-Related Apoptosis-Inducing Ligand (TRAIL) is an endogenous ligand that is expressed in various immune cells. TRAIL mediates apoptosis through binding of transmembrane receptors, death receptor 4 (DR4) and/or death receptor 5 (DR5). Cancer cells are frequently resistant to TRAIL-mediated apoptosis, and the cause of this may be decreased expression of death receptors. This study aimed to identify combination therapies that exploit XN for NB. First, the effect of XN on cellular proliferation in human NB cell lines NGP, SH-SY-5Y, and SK-N-AS were determined via MTT assay, colony forming assay, and real-time live cell imaging confluency. XN treatment causes a statistically significant decrease in the viability of NB cells with IC50 values of approximately 12 μM for all three cell lines. Inhibition of cell proliferation via apoptosis was evidenced by an increase in pro-apoptotic markers (cleaved PARP, cleaved caspase-3/-7, and Bax) and a decrease in an anti-apoptotic marker, Bcl-2. Importantly, XN treatment inhibited PI3K/Akt pathway and associated with increased expression of DR5 by both mRNA and protein levels. Furthermore, a statistically significant synergistic reduction was observed following combination treatment (50%) compared to either TRAIL (5%) or XN (15%) alone in SK-N-AS cells. Therefore, this study shows XN treatment reduces NB cell growth via apoptosis in a dose-dependent manner, and enhanced growth reduction was observed in combination with TRAIL. This is the first study to demonstrate that XN alters the expression of DR5 as well as the synergistic effect of XN on TRAIL in NB and provides a strong rationale for further preclinical analysis.

## Introduction

Neuroblastoma (NB) is a devastating cancer of the sympathetic nervous system that predominantly strikes children [1, 2]. Young children have a better outcome due to a higher rate of spontaneous regression and are more amenable to current therapies. However, nearly all patients over 18 months of age relapse, especially those with high-risk features, such as advanced stage, chemoresistance, relapsed disease, and amplification of MYCN [1–6]. MYCN is an oncogene that produces the N-myc protein and is associated with high-risk NB [7]. Relapsed NB is extremely difficult to cure, as it is notoriously resistant to traditional modalities, so new therapeutic strategies and adjunctive compounds are integral [8].

**Fig 1:**
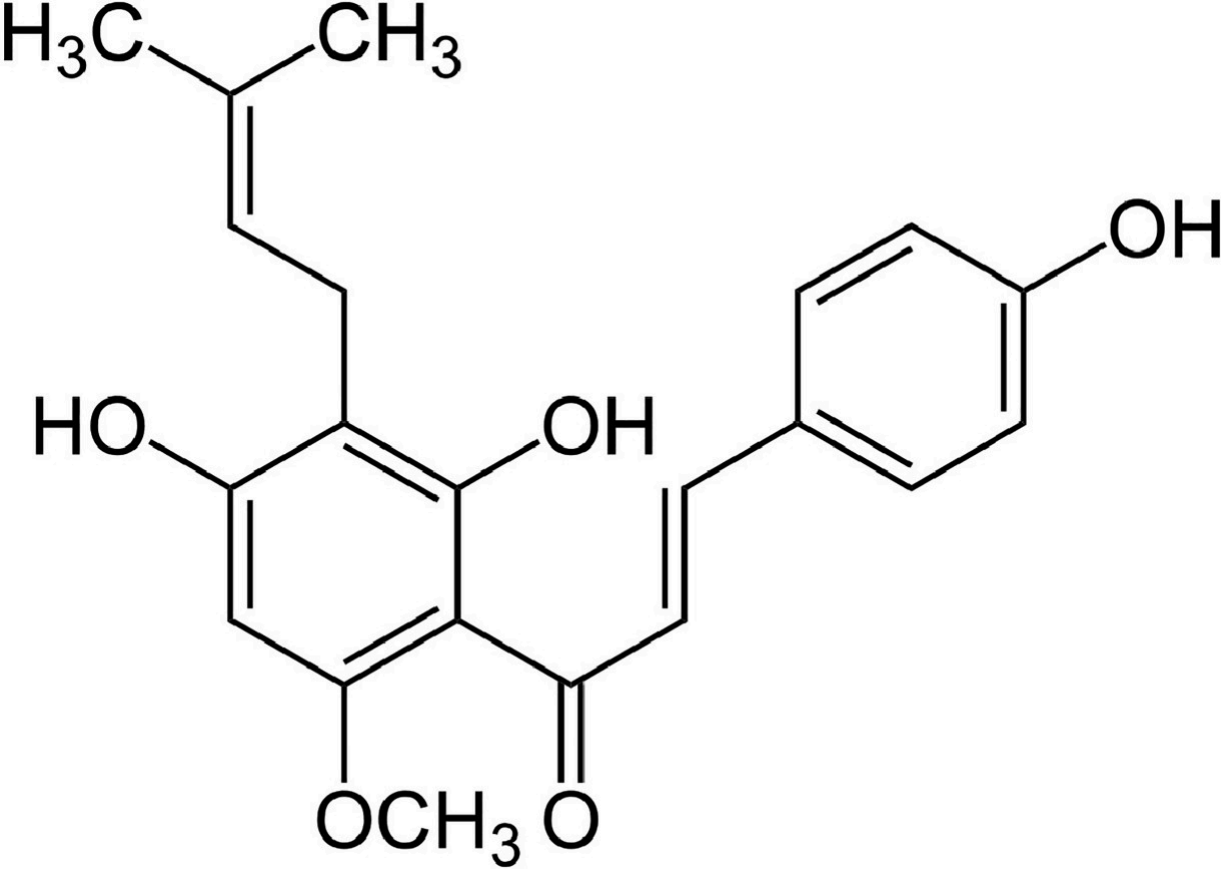
Chemical structure of Xanthohumol.

Utilizing natural products as lead compounds is a useful and practical method in drug development. One such compound, Xanthohumol (XN) (Fig 1), a prenylated chalcone found in hops, inhibits tumor cell proliferation and angiogenesis, and induces apoptosis in a variety of cancer cells [9–13]; however, the mechanism by which XN functions is not well understood [12, 14–19]. Our recent publications demonstrate XN activity against pancreatic, hepatocellular, and cholangiocarcinoma *in vitro* [10, 11, 13]. Currently, a phase I clinical trial is ongoing to test XN activity in humans (NCT 02432651). A single dose pharmacokinetic study in humans identified XN in plasma with a mean half-life of 18 and 20 hours for the 60 and 180 mg doses, respectively [20], by liquid-chromatography tandem mass spectrometry [21, 22]. Furthermore, oral administration of XN (50 μg/mouse, approximately 2.5 mg/kg) delayed tumor progression and reduced the cell growth of poorly differentiated prostate carcinoma in transgenic mice containing adenocarcinoma of the mouse prostate (TRAMP) [12]. The concentration of XN used for *in vitro* studies on prostate cancer cells was between 20–40 μM [12, 15, 23]. Recently, we reported that there was a delay in tumor progression in cholangiocarcinoma xenograft after XN treatment [13]. Another agent of interest is the TNF-related apoptosis-inducing ligand (TRAIL) cytokine which is expressed in various immune cells including CD4^+^ T cells, NK cells, macrophages, and dendritic cells and binds to death receptor 5 (DR5) to induce apoptosis [24]. This receptor is considered part of the extrinsic as well as the intrinsic pathway of apoptosis [25–27]. Several reports have suggested that highly malignant N-type NB cell lines are resistant to TRAIL-mediated cell death, whereas more differentiated and noninvasive S-type NB cell lines remain susceptible to TRAIL [28–30]. Up-regulation of DR5 is important for sensitivity to TRAIL-induced apoptosis and is a transcriptional target of p53 [31]. Deletion of DR5 causes resistance to TRAIL-mediated apoptosis as well as an abrogated response to DNA damaging stimuli, while induction of DR5 promotes cancer cell death. It was suggested that the activation of AKT may also contribute to the development of TRAIL resistance in prostate cancer cells [32, 33]. Both XN and TRAIL have therapeutic potential, and this study looks at the effect of these compounds in NB cell lines.

We have previously shown the effect of XN on a series of organ-specific tumors *in vitro*; however, the effect of XN on TRAIL-induced apoptosis is not fully understood. Here, we sought to investigate the role of XN on a panel of NB cell lines and demonstrate that XN sensitizes NB cells to TRAIL-induced apoptosis through DR5 expression. Therefore, we believe XN and TRAIL have a synergistic effect on NB cell lines.

## Materials and methods

### Cell culture and reagents

Three human neuroblastoma (NB) cell lines (SK-N-AS, NGP, and SH-SY-5Y) were used in this study, and SK-N-AS and SH-SY-5Y were purchased from ATCC. The NGP was a kind gift from Dr. Thiele. The cells were grown in Roswell Park Memorial Institute Media (RPMI; Life Technologies, Carlsbad, CA), supplemented with 10% fetal bovine serum (Sigma-Aldrich, St. Louis, MO), 100 IU/mL penicillin, and 100 μg/mL streptomycin (Life Technologies) in a humidified atmosphere of 5% CO_2_ in air at 37°C. Xanthohumol (Tocris, Minneapolis, MN or Selleckchem, Houston, TX) was dissolved in dimethyl sulfoxide (DMSO: Sigma-Aldrich) to prepare stock solutions of 50 mM. An equivalent volume of DMSO alone served as a negative control. TRAIL/Apo-2L was purchased from PeproTech (Rocky Hill, New Jersey, USA) and stored in aliquots at -80°C.

### Cellular proliferation assays

NB cell proliferation was measured using a 3-(4, 5-dimethylthiazole-2-yl)-2, 5-diphenyl tetrazolium bromide (MTT) (Sigma-Aldrich, St. Louis, MO) assay per manufacturer instructions and our earlier reports [10, 11, 13]. Cells were seeded in 96-well plates and treated with different concentrations of XN for up to 96 hours. For combination study of XN and TRAIL cells were incubated for 48 hours. The results represent the average of three experiments, each conducted in quadruplicate. Values were calculated to percent growth relative to vehicle control (0.1% DMSO). Statistical analysis was performed using the online software IBM SPSS (IBM, North Castle, NY). Unpaired t-tests were performed on each data set and P-values <0.05 were considered significant.

### Colony formation assay

To further assess the ability of XN to reduce cellular proliferation, the colony-forming ability was determined by the measurement of the colonogenic cell survival. Cells were plated onto 6-well plates and treated with various concentrations of XN for 3 days. Then, media was removed and replaced with XN-free media. Following one week, media was again removed, and the cells were fixed with crystal violet staining and photographs were taken using Molecular Imager ChemiDoc XRS+ imager with image lab software (Bio-Rad, Hercules, CA). Colonies were then counted and compared to control.

### Noninvasive cellular proliferation assay in real-time

Using IncuCyte Live-Cell Imaging systems (Essen Bioscience, Ann Arbor, MI), cellular proliferation of NB cancer cell lines was measured as previously described [34]. Briefly, SK-N-AS (2000 cells/well), NGP (2000 cells/well), and SH-SY-5Y (5000 cells/well) were plated onto a 96-well plate and incubated at 37°C for 12 hours. The cells were then treated with varying concentrations of XN (0–30 μM) for up to 96 hours. The cells were imaged every 2 hours using a 10× objective for the duration of the incubation. Cell confluence was calculated using IncuCyte 2011A (Essen Bioscience Inc, Ann Arbor, MI) software. The cell proliferation was expressed and graphed as an increase in confluence percentage as described [10, 11, 13].

### Cell player YOYO-1 cytotoxicity assay

This assay is based on the membrane integrity of the cells and the use of YOYO-1 iodide (491/509) (Life Technologies), 491/509, a fluorescent dye and CellPlayer Kinetic Caspase3/7 Apoptosis reagent (Life Technologies). Cells were plated in 96-well plates and incubated overnight. Then the cells were treated with various concentrations of xanthohumol, YOYO-1 (1:10000), and CellPlayer (1:10000), and continued to incubate in an Incucyte for 96 hours. Images of both phase-contrast and fluorescence were collected every 2–4 hours and analyzed using IncuCyte 2011A (Essen Bioscience Inc, Ann Arbor, MI) software.

### Analysis of apoptosis by flow cytometry

Apoptosis assay was carried out using Annexin V-FITC Apoptosis Detection Kit (Sigma) as described by the manufacturer. Briefly, XN treated cells were collected and washed with PBS. Then the pellet was suspended in binding buffer, 3 μl Annexin V-FITC and 5 μl of PI from the kit for 15 min in dark. Finally, the cells were analyzed using Galios, Beckman-Coulter flow cytometry. The data were analyzed using Kaluza flow cytometry analysis V1.2 software. Each experiment was performed in triplicates and repeated once.

### Western analysis

NB cell lysates were isolated following 3 days of XN-treatment (0–15μM) using the radioimmunoprecipitation assay buffer (RIPA; Thermo Fischer Scientific). Denatured cellular extracts (20 μg) were boiled with equal volumes (1:1) of loading dye (2% sodium dodecyl sulfate, 20% glycerol, 0.1mol/L Tris 5, 13-mercaptoethanol, 0.04% bromophenol blue) for 5 min and electrophoresed through 7.5%, 10%, and/or 12% Mini-Protean TGX gels (Bio-Rad Laboratories). The proteins were then transferred onto nitrocellulose membranes (Bio-Rad Laboratories), blocked in milk solution (5% dry skim milk and 0.05% Tween-20 in 1X PBS) for 1 h, and incubated in primary antibody overnight at 4°C.

Antibodies against GAPDH (1:5000), Bax (1:200), cyclinD1 (1:500), and cleaved PARP (1:1000) were purchased from Santa Cruz Biotechnology and cleaved caspase-3 (1:1000), DR5 (1:1000), Bcl-2 (1:1000), and phosphorylated AKT ser 473 (1:1000) were obtained from Cell Signaling Technology Inc., respectively. Following overnight incubation, membranes were washed thrice in a wash buffer containing 1x phosphate buffered solution containing 0.05% Tween-20 buffer. Following the successful wash, the membranes were incubated in either HRP-conjugated goat anti-rabbit IgG antibody (1:10000 dilution) or goat anti-mouse IgG antibody (1:10000 dilution) for 90 minutes. After incubation, the membranes were washed 3 X 5 times with wash buffer. The proteins were then visualized using SuperSignal West Femto (Pierce), Immun-Star (Bio-Rad), or Dura (Pierce) kits per manufacturer instructions. The membranes were then imaged to capture the band intensity using the Molecular Images Chemi-Doc XRS^+^ imager (Bio-Rad).

### Detection of DR5 mRNA expression

Reverse transcription-PCR was conducted to quantify DR5 gene expression using mRNA from control or XN-treated cells as previously described [11] using GAPDH (glyceraldehyde 3-phosphate dehydrogenase) as internal control mRNA. DR5, 5′-GACCTAGCTCCCCAGCAGAGAG-3′ (sense) and 5′-CGGCTGCAACTGTGACTCCTAT-3′ (antisense) and GAPDH; forward 5′-ACCTGCCAAATATGATGAC-3′ and reverse 5′-ACCTGGTGCTCAGTGTAG-3′) were used for RT-PCR.

### Caspase-3 and -7 activities

Caspase-Glo 3/7 Assay kit (Promega) was used to measure the cleaved caspase-3 and -7 activities from cell lysates after xanthohumol treatment as described previously [11]. Following drug treatment and cell lysis, a luminogenic caspase-3/7 substrate containing the tetrapeptide sequence DEVD was added to the cell lysate. Then the luminescence was measured in triplicate samples using Infinite M200PRO Microplate reader (TECAN).

### Statistical analysis

One-way ANOVA analysis was performed using a statistical analysis software package (IBM SPSS Statistics version 22). A p value of <0.05 was considered significant. Data was represented as + SE.

## Results

### Xanthohumol treatment suppresses neuroblastoma cell growth

**Fig 2:**
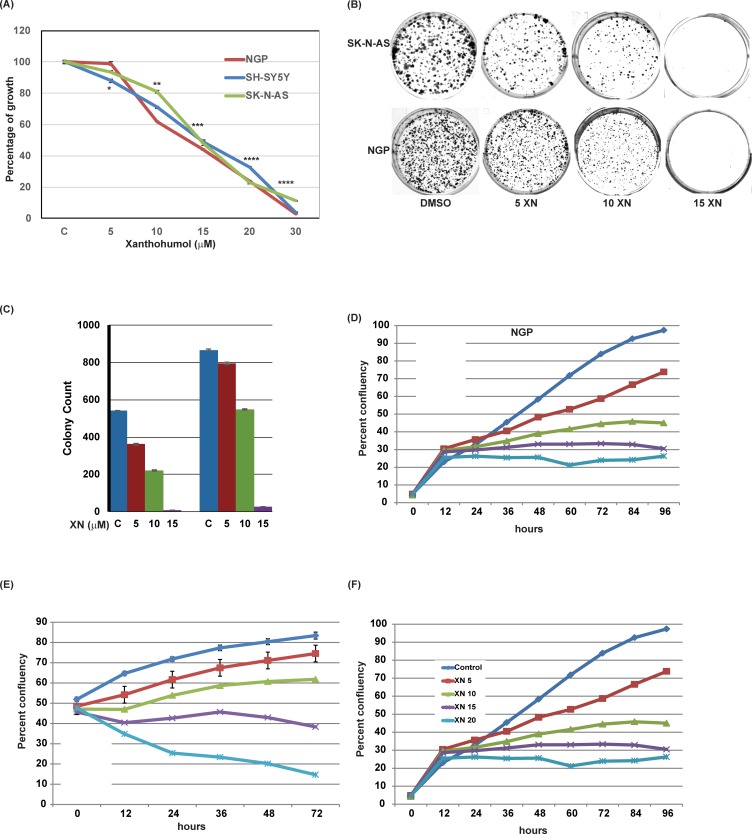
Effects of XN on NGP, SH-SY-5Y, and SK-N-AS cellular proliferation. MTT assay **(A)**, Colony forming unit **(B-C),** and IncuCyte Live-Cell imaging **(D-F)** demonstrate a dose-dependent reduction in cell growth compared to control. * p<0.05; ** p<0.001; *** p<0.0001.

Cellular growth of NB cell lines was determined via MTT assay. As shown in Fig 2A, there was a dose-dependent suppression of cellular growth in all three NB cell lines compared to vehicle (DMSO) controls after 3 days of treatment. The IC_50_ value was approximately 12 μM (P ≤ 0.01) for all cell lines. To confirm the growth suppression by XN, colony forming assay was performed (Fig 2B). In SK-N-AS and NGP cell lines, there was a significant reduction in colony formation with increasing concentrations of XN, beginning at concentrations greater than 10 μM which is depicted as a bar graph (Fig 2C).

Live cell imaging was also used to further demonstrate the role of XN on cellular proliferation. Live cell image assay can be used as a surrogate marker for cellular proliferation, as this allows us to calculate the confluency. When compared to control (DMSO), cellular growth (Fig 2D–2F) is reduced in each NB cell line in a dose-dependent manner.

### Xanthohumol induces apoptosis in neuroblastoma cells

**Fig 3:**
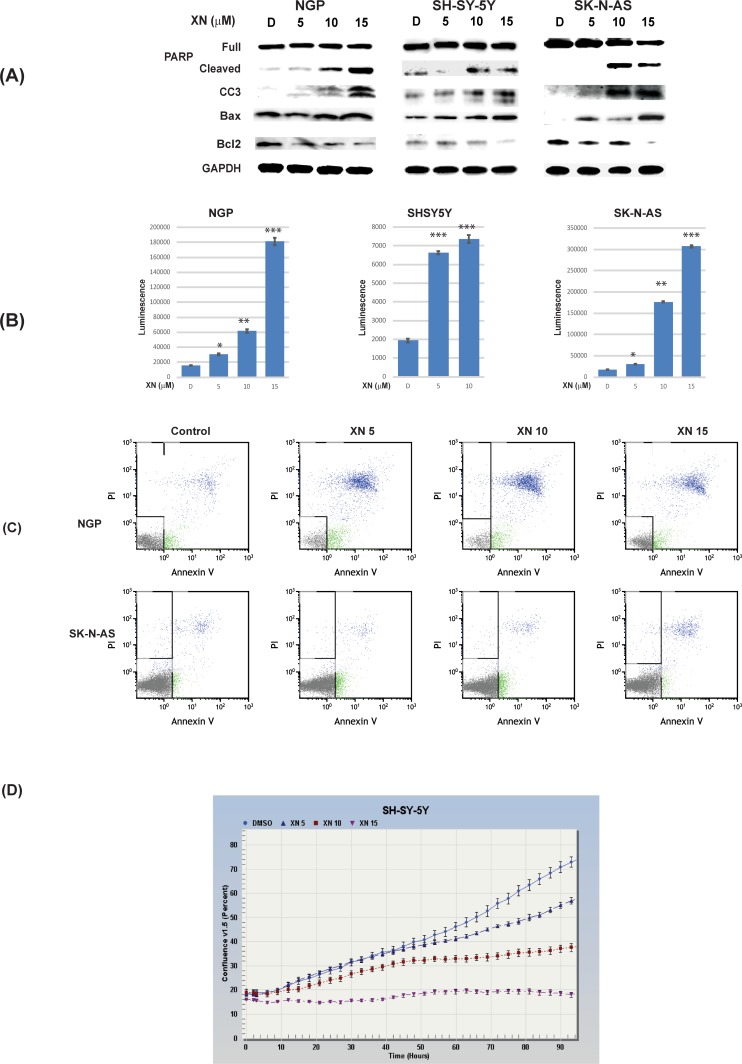
XN induces apoptosis in NGP, SH-SY-5Y, and SK-N-AS cells. **A.** Western blot analysis showed an increase in apoptotic markers, cleaved PARP, cleaved caspase 3 (CC3) and Bax after treatment with XN for 72hrs. This was associated with a reduction in anti-apoptotic protein, Bcl-2. **B.** Caspase-3/-7 activities increased in a dose-dependent fashion in XN-treated NGP, SK-N-AS, and SH-SY-5Y cells. * p<0.05; ** p<0.001; *** p<0.0001. **(C).** Annexin-V-FITC flow cytometry analysis showed an increase in apoptosis. **(D).** Live cell image of cell player with YOYO-1 assay by Incucyte also showed induction of apoptosis.

We have shown that XN treatment induces apoptosis in other cancer types [10, 11, 13]. Therefore, to investigate the mechanism by which XN inhibited cellular growth, Western analyses were performed to check the levels of apoptosis-related markers. As seen in Fig 3A, XN treatment induced the expression of pro-apoptotic markers cleaved caspase-3, cleaved PARP, and Bax in all three cell lines in a dose-dependent fashion. Additionally, XN treatment decreased the expression of anti-apoptotic protein Bcl-2 in all three NB cell lines. Cleaved caspase-3 and -7 activities were increased with XN treatment in all three cell lines confirming the apoptotic induction (Fig 3B). This was further confirmed by Annexin V-FITC flow cytometry (Fig 3C), and the measurement of kinetic activation of Caspase3/7 (Fig 3D). These findings suggest that XN’s antiproliferative effect on NB is through apoptosis, consistent with previous studies in other cancer types.

### Xanthohumol inhibits PI3K/Akt pathway and increases DR5 expression in neuroblastoma cells

**Fig 4:**
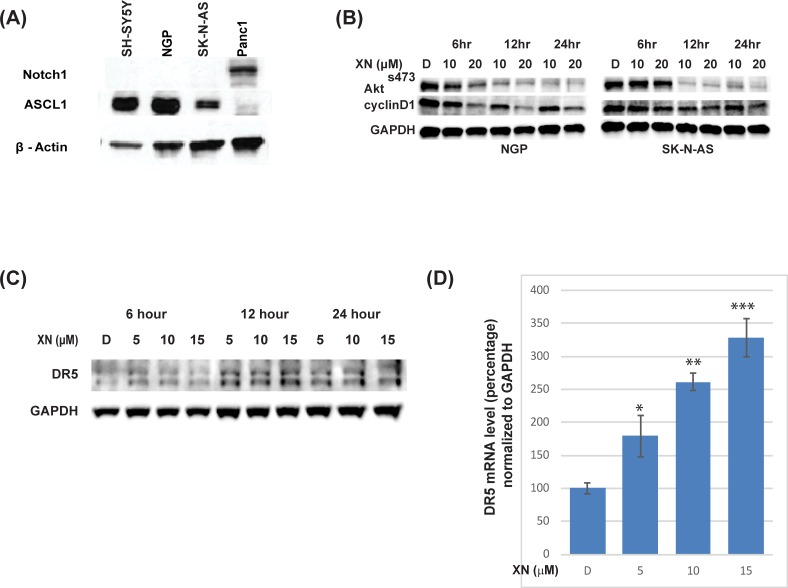
XN regulates the Akt pathway and associated with an increase in DR5 expression. **A.** Notch signaling is minimally active in NGP, SK-N-AS, and SH-SY-5Y, as evidenced by minimal expression of Notch intracellular Domain1, whereas there is increased expression of achaete-scute complex-like1 (ASCL1), a downstream target of Notch signaling. B. Inhibition of Akt pathway by XN treatment was observed by reduction in phosphorylation of Akt (ser473) as early as 6 hrs. This was associated with a reduction in CyclinD1. **C.** NB cell line, SK-N-AS was treated with indicated concentrations of XN for 6–24 hours’ time points and lysates were prepared. Western blot analysis of the lysates shows a time-dependent increase in DR5 expression following XN treatment. GAPDH was used as loading control. **D.** DR5 gene expression increases with XN treatment in a dose-dependent fashion in quantitative RT PCR analysis. GAPDH was used as a control and for normalization. * p<0.05; ** p<0.01; *** p<0.001.

In our earlier studies, we have shown that growth reduction is in part by the reduction in Notch1 protein [10, 11, 13]; however, recent work on Notch1 signaling on differentiation and malignancies in NB are conflicting [35, 36]. Therefore, we determined the expression of Notch1 in NB cell lines used in this study. As shown in Fig 4A, active Notch1 (Notch intracellular Domain-NICD1) protein is minimally present in all three cell lines used in the study. As a comparison, we used pancreatic cancer cell line, Panc1 as a positive control. It is interesting to note that the Notch target gene, human achaete-scute complex-Like1 (ASCL1), is expressed only in these cell lines where Notch1 is not expressed, indicating that Notch1 signaling is minimally active in these cell lines. Inhibition of the PI3K/Akt pathway is an important target strategy as this pathway is highly expressed in neuroblastoma [37, 38]. It has been shown that the PI3K/AKT signaling pathway plays an oncogenic role in SH-SY-5Y cells [39, 40]. To determine if xanthohumol inhibits this pathway, we carried out western blot analysis using lysates from XN-treated NB cells. Results showed a reduction in the phosphorylation of AKT ser 473 as early as 6 hours after XN treatment in both NGP and SK-N-AS cells (Fig 4B). This was associated with a reduction in cyclinD1, a downstream target of AKT pathway.

Since these cell lines are resistant to TRAIL treatment, we were interested to determine if DR5 plays a role in XN treatment. Further analysis of XN’s effect on NB was carried out through Western analyses. DR5 expression levels were determined in SK-N-AS cells treated with XN at different time intervals (6 hours, 12 hours, and 24 hours). Fig 4C shows an increase in DR5 receptor expression following XN treatment, primarily after 12 hours at a concentration of 10–15 μM. To determine the increase in protein levels is due to increase in DR5 mRNA, we carried out quantitative real-time PCR after XN treatment and the result shows an increase in DR5 gene expression, indicating a transcriptional mechanism of regulation (Fig 4D).

### Synergism between XN and TRAIL

**Fig 5:**
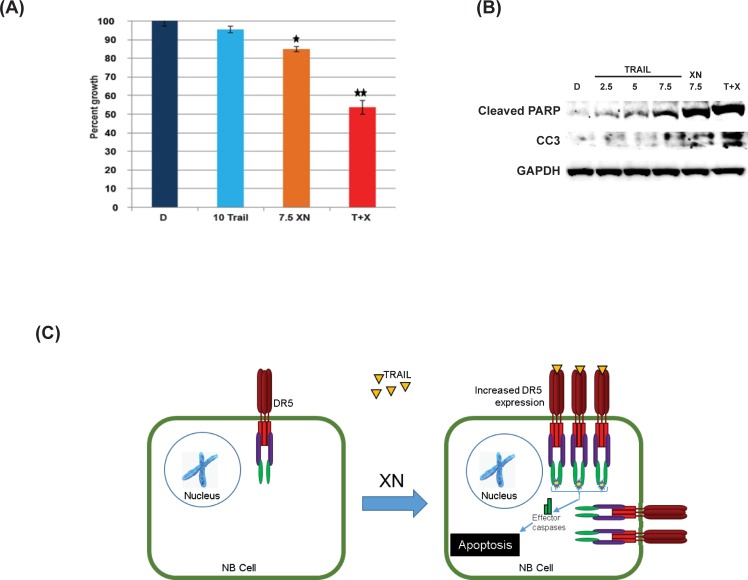
Effects of XN and TRAIL treatment after 48hrs on SK-N-AS cellular proliferation. MTT assay **(A)** and Western blot analysis **(B)** showed synergism between XN and TRAIL on cell viability and increased apoptotic marker expression (cleaved PARP and CC3). * P-value <0.05; **0.005. **C.** A proposed mechanism of synergistic effects of XN and TRAIL on NB cells. XN increases DR5 expression, which increases the probability of TRAIL binding to DR5, thereby inducing apoptosis.

Since XN induces apoptosis and is associated with an increase in DR5 expression, we wanted to determine if XN sensitizes cells to TRAIL treatment in combination with TRAIL ligand. For this, the effect of XN and TRAIL combination treatment of SK-N-AS cells was evaluated via MTT assay and further by western analysis. In Fig 5A, treatment with 10 ng of TRAIL and 7.5 μM XN alone for 2 days resulted in approximately 5% (P ≤ 0.05) and 15% (P ≤ 0.05) reductions in growth, respectively. Combination treatment with XN (7.5 μM) and TRAIL (10 ng) demonstrated an approximate growth reduction of 50% (P ≤ 0.01). To determine the synergistic effect of this combination, we calculated synergy as a percent in the observed response over the expected response. The calculated combination index was 41% (0.41). Expression of cleaved PARP and cleaved caspase-3 is significantly increased for combination treatment of XN (7.5 μM) and TRAIL (10 ng) when compared to XN (7.5 μM) or TRAIL (2.5 ng, 5 ng, and 7.5 ng) alone (Fig 5B). These data demonstrate potential synergism between XN and TRAIL in treating NB.

## Discussion

Neuroblastoma is a common childhood malignancy and is responsible for a significant portion of pediatric cancer deaths. One of the primary treatments for this is surgical resection, especially for the low and intermediate risk tumors, which occur in adolescents and adults. For high-risk, metastatic, and/or recurrent tumors, aggressive treatment with chemotherapy, high-dose chemotherapy with hematopoietic stem-cell rescue, surgical resection, and radiation proves ineffective. Therefore, a novel therapy to treat these patients is much needed.

Xanthohumol (XN), a flavonoid compound isolated from the hop plant, *Humulus lupulus*, has been shown to have significant efficacy against breast, prostate, ovarian, thyroid, colon, pancreas, bile duct, and hepatocellular cancer cells [10–13, 16–18, 23, 41]. The pharmacologic characteristics of XN include anti-obesity, hypoglycemic, anti-hyperlipidemia, cancer chemo-preventive, anti-angiogenic, anti-invasion, anti-microbial, anti-parasite, and hepatoprotective activities, among many more. Additionally, toxicology studies of XN in animals have shown that XN did not affect major organ functions [42, 43]. XN has also been evaluated in menopausal women and had no acute toxicity was seen [44]. Despite the growing evidence of the efficacy of XN, it still has not been thoroughly analyzed in NB. We observed a significant reduction in cellular viability and growth in three genetically diverse NB cell lines treated with XN. These cell lines have different genetic groups; SH-SY-5Y (Non-amplified MYCN or single copy, TP53 WT, ALK mutation (F1174L)), NGP 1p alteration (t(1p), MYCN amplified, ALK wild type, TP53 mutated-MDM2 amplified), and SK-N-AS (1p deletion, MYCN single copy, TP53 mutation (H168R), and ALK WT). Western analysis, caspase-3/-7 luminescent studies, apoptosis assay flow cytometry, and YOYO-1 fluorescent assays showed that XN induces apoptosis in NB cell lines tested.

We have recently reported that XN treatment reduces growth and it is partly due to the reduction in Notch1 signaling [10, 11, 13]. However, the role of Notch1 signaling in NB is contradictory and conflicting. It was reported that Notch1 expression showed unfavorable prognosis in NB tumors. In this report, however, they showed positive Notch expression that includes a weak expression (10–35% cells stained), moderate expression (35–70% cell stained) and strong expression (more than 70% cells stained) was only about 50% [35]. This result indicated that the remaining 50% of the tumors were negative for Notch1 expression. We observed that active Notch1 (Notch intracellular Domain-NICD1) is lacking or minimally present in SH-SY5Y, NGP and SK-N-AS cell lines tested, indicating that the mechanism of growth suppression by XN in these cells may be by altering another oncogenic pathway. Therefore, we looked at another dominant oncogenic pathway, PI3K/AKT, that has been highly activated in NB. Activation of AKT phosphorylation increases its downstream target proteins that are involved in cell cycle progression including Cyclin D1, and pro-apoptotic protein including Bcl2. XN has been shown to inhibit the Akt pathway at a higher concentration in prostate cancer both in vitro and in vivo [12]. Here, we show that in NB cell line, XN inhibited the Akt activation as evidenced by the reduction in phosphorylation of AKT ser 473 and its downstream target Cyclin D1, TNF-related apoptosis-inducing ligand (TRAIL) is a cytokine that induces apoptosis via binding to death receptor 5 (DR5) [24]. It has been shown that various cancers including NB, pancreatic cancer, and melanoma are resistant to TRAIL-mediated apoptosis [45–47]. We believe that down-regulation of DR5 is a potential mechanism of resistance to TRAIL treatment as it is frequently associated with silencing by hypermutation, deletions, or point mutations [48–51]. Western analysis showed an increase in DR5 expression in SK-N-AS cells treated with XN after 12–24 hours, and RT-PCR analysis showed an increase in DR5 gene expression in XN treated SK-N-AS cells. To determine whether this increase in DR5 expression sensitizes NB cells to TRAIL, we looked at a combination of XN and TRAIL. Treatment with XN followed by TRAIL showed a synergistic reduction in cellular growth in MTT assay. Western analysis showed a significant increase in apoptotic markers, cleaved PARP, and cleaved caspase-3.

*In vivo*, XN has been shown to reduce the growth of poorly differentiated prostate tumors without adverse side effects [12], which demonstrates that XN may be a novel agent for the management of solid organ tumor. Recently, XN metabolism and pharmacokinetics parameters were tested (NCT01367431) [20]. We believe that the concentrations of XN used in our study could be achieved in humans with minimal side effects. However, phase I dose-escalation studies are still needed to determine the maximum tolerable dose and potential adverse effects in humans.

## Conclusions

We believe our research findings are significant for demonstrating a potential therapy to treat NB, and there is a strong rationale to further study these agents in NB, both *in vitro* and *in vivo*. We have shown evidence of synergism between XN and TRAIL in NB cells, with a proposed mechanism seen in Fig 5C. Our study supports the hypothesis that an increase in DR5 increases the probability of TRAIL binding and inducing apoptosis. Although the precise molecular mechanism driving the expression of DR5 and the inhibition of the AKT pathway in response to XN treatment needs further exploration, this is beyond the scope of this study. These studies, however, do support further exploration of therapeutic strategies based on these combinations with XN and other chemotherapeutic agents in NB and other cancers. Furthermore, effective combination therapies would induce apoptosis rather than just suppress growth, and this could possibly be mitigating dose limitation as well as other toxicities. In summary, this study will provide new opportunities to discover effective combination as well as additional targets in XN treatment that may be more effective in various tumor types.

## References

[1] Brodeur GM, Bagatell R. Mechanisms of neuroblastoma regression. Nature reviews Clinical oncology. 2014;11(12):704–13. 10.1038/nrclinonc.2014.168 25331179 PMC4244231

[2] Phillips SM, Padgett LS, Leisenring WM, Stratton KK, Bishop K, Krull KR, et al Survivors of childhood cancer in the United States: prevalence and burden of morbidity. Cancer epidemiology, biomarkers & prevention: a publication of the American Association for Cancer Research, cosponsored by the American Society of Preventive Oncology. 2015;24(4):653–63. 10.1158/1055-9965.EPI-14-1418 25834148 PMC4418452

[3] Suganuma R, Wang LL, Sano H, Naranjo A, London WB, Seeger RC, et al Peripheral Neuroblastic Tumors with Genotype-Phenotype Discordance: A Report from the Children’s Oncology Groupand the International Neuroblastoma Pathology Committee. Pediatric blood & cancer. 2013;60(3):363–70. 10.1002/pbc.24238 ; PubMed Central PMCID: PMCPmc3397468. 22744966 PMC3397468

[4] Teshiba R, Kawano S, Wang LL, He L, Naranjo A, London WB, et al Age-Dependent Prognostic Effect by Mitosis-Karyorrhexis Index in Neuroblastoma: A Report from the Children’s Oncology Group. Pediatric and developmental pathology: the official journal of the Society for Pediatric Pathology and the Paediatric Pathology Society. 2014;17(6):441–9. 10.2350/14-06-1505-oa.1 ; PubMed Central PMCID: PMCPmc4340697. 25207821 PMC4340697

[5] Wang LL, Suganunma R, Ikegaki N, Tang X, Naranjo A, McGrady P, et al Neuroblastoma—Undifferentiated Subtype, Prognostic Significance of Prominent Nucleolar Formation and MYC/MYCN Protein Expression: A Report from the Children’s Oncology Group. Cancer. 2013;119(20):3718–26. 10.1002/cncr.28251 PMC4554323. 23901000 PMC4554323

[6] Wang LL, Teshiba R, Ikegaki N, Tang XX, Naranjo A, London WB, et al Augmented expression of MYC and/or MYCN protein defines highly aggressive MYC-driven neuroblastoma: a Children/'s Oncology Group study. Br J Cancer. 2015;113(1):57–63. 10.1038/bjc.2015.188 26035700 PMC4647535

[7] Richards MW, Burgess SG, Poon E, Carstensen A, Eilers M, Chesler L, et al Structural basis of N-Myc binding by Aurora-A and its destabilization by kinase inhibitors. 2016 . 27837025 10.1073/pnas.1610626113 PMC5137718

[8] Huang M, Weiss WA. Neuroblastoma and MYCN. Cold Spring Harbor perspectives in medicine. 2013;3(10):a014415 10.1101/cshperspect.a014415 24086065 PMC3784814

[9] Dorn C, Weiss TS, Heilmann J, Hellerbrand C. Xanthohumol, a prenylated chalcone derived from hops, inhibits proliferation, migration and interleukin-8 expression of hepatocellular carcinoma cells. International journal of oncology. 2010;36(2):435–41. . 20043079

[10] Kunnimalaiyaan S, Sokolowski KM, Balamurugan M, Gamblin TC, Kunnimalaiyaan M. Xanthohumol inhibits Notch signaling and induces apoptosis in hepatocellular carcinoma. PloS one. 2015;10(5):e0127464 10.1371/journal.pone.0127464 26011160 PMC4444108

[11] Kunnimalaiyaan S, Trevino J, Tsai S, Gamblin TC, Kunnimalaiyaan M. Xanthohumol-Mediated Suppression of Notch1 Signaling Is Associated with Antitumor Activity in Human Pancreatic Cancer Cells. Molecular cancer therapeutics. 2015;14(6):1395–403. 10.1158/1535-7163.MCT-14-0915 25887885 PMC4554525

[12] Vene R, Benelli R, Minghelli S, Astigiano S, Tosetti F, Ferrari N. Xanthohumol impairs human prostate cancer cell growth and invasion and diminishes the incidence and progression of advanced tumors in TRAMP mice. Molecular medicine. 2012;18:1292–302. 10.2119/molmed.2012.00174 22952060 PMC3521786

[13] Walden D, Kunnimalaiyaan S, Sokolowski K, Gamblin TC, Kunnimalaiyaan M. Antiproliferative and apoptotic effects of xanthohumol in cholangiocarcinoma. Oncotarget. 2017;8(50):88069–78. 10.18632/oncotarget.21422 WOS:000413341400086. 29152142 PMC5675694

[14] Albini A, Dell'Eva R, Vene R, Ferrari N, Buhler DR, Noonan DM, et al Mechanisms of the antiangiogenic activity by the hop flavonoid xanthohumol: NF-kappaB and Akt as targets. FASEB J. 2006;20(3):527–9. 10.1096/fj.05-5128fje 16403733

[15] Colgate EC, Miranda CL, Stevens JF, Bray TM, Ho E. Xanthohumol, a prenylflavonoid derived from hops induces apoptosis and inhibits NF-kappaB activation in prostate epithelial cells. Cancer letters. 2007;246(1–2):201–9. 10.1016/j.canlet.2006.02.015 . 16563612

[16] Cook MR, Luo J, Ndiaye M, Chen H, Kunnimalaiyaan M. Xanthohumol inhibits the neuroendocrine transcription factor achaete-scute complex-like 1, suppresses proliferation, and induces phosphorylated ERK1/2 in medullary thyroid cancer. American journal of surgery. 2010;199(3):315–8; discussion 8. 10.1016/j.amjsurg.2009.08.034 20226902 PMC2841322

[17] Delmulle L, Vanden Berghe T, De Keukeleire D, Vandenabeele P. Treatment of PC-3 and DU145 prostate cancer cells by prenylflavonoids from hop (Humulus lupulus L.) induces a caspase-independent form of cell death. Phytother Res. 2008;22(2):197–203. 10.1002/ptr.2286 WOS:000254229000010. 17726738

[18] Pan L, Becker H, Gerhauser C. Xanthohumol induces apoptosis in cultured 40–16 human colon cancer cells by activation of the death receptor- and mitochondrial pathway. Molecular nutrition & food research. 2005;49(9):837–43. 10.1002/mnfr.200500065 . 15995977

[19] Vanhoecke B, Derycke L, Van Marck V, Depypere H, De Keukeleire D, Bracke M. Antiinvasive effect of xanthohumol, a prenylated chalcone present in hops (Humulus lupulus L.) and beer. International journal of cancer Journal international du cancer. 2005;117(6):889–95. 10.1002/ijc.21249 . 15986430

[20] Legette L, Karnpracha C, Reed RL, Choi J, Bobe G, Christensen JM, et al Human pharmacokinetics of xanthohumol, an antihyperglycemic flavonoid from hops. Molecular nutrition & food research. 2014;58(2):248–55. Epub 2013/09/17. 10.1002/mnfr.201300333 24038952 PMC4371792

[21] Legette L, Ma L, Reed RL, Miranda CL, Christensen JM, Rodriguez-Proteau R, et al Pharmacokinetics of xanthohumol and metabolites in rats after oral and intravenous administration. Molecular nutrition & food research. 2012;56(3):466–74. 10.1002/mnfr.201100554 22147307 PMC3401605

[22] Legette LL, Luna AY, Reed RL, Miranda CL, Bobe G, Proteau RR, et al Xanthohumol lowers body weight and fasting plasma glucose in obese male Zucker fa/fa rats. Phytochemistry. 2013;91:236–41. 10.1016/j.phytochem.2012.04.018 . 22640929

[23] Deeb D, Gao X, Jiang H, Arbab AS, Dulchavsky SA, Gautam SC. Growth inhibitory and apoptosis-inducing effects of xanthohumol, a prenylated chalone present in hops, in human prostate cancer cells. Anticancer research. 2010;30(9):3333–9. . 20944105 PMC3846352

[24] Di Pietro R, Zauli G. Emerging non-apoptotic functions of tumor necrosis factor-related apoptosis-inducing ligand (TRAIL)/Apo2L. Journal of cellular physiology. 2004;201(3):331–40. Epub 2004/09/25. 10.1002/jcp.20099 . 15389537

[25] Wu X, Yang N, Zhou WH, Xu J, Chen JJ, Zheng FM, et al Up-regulation of P21 inhibits TRAIL-mediated extrinsic apoptosis, contributing resistance to SAHA in acute myeloid leukemia cells. Cellular physiology and biochemistry: international journal of experimental cellular physiology, biochemistry, and pharmacology. 2014;34(2):506–18. 10.1159/000363018 . 25116350

[26] Song JH, Song DK, Pyrzynska B, Petruk KC, Van Meir EG, Hao C. TRAIL triggers apoptosis in human malignant glioma cells through extrinsic and intrinsic pathways. Brain pathology. 2003;13(4):539–53. . 14655759 10.1111/j.1750-3639.2003.tb00484.x PMC8096004

[27] Park MR, Kim SG, Cho IA, Oh D, Kang KR, Lee SY, et al Licochalcone-A induces intrinsic and extrinsic apoptosis via ERK1/2 and p38 phosphorylation-mediated TRAIL expression in head and neck squamous carcinoma FaDu cells. Food and chemical toxicology: an international journal published for the British Industrial Biological Research Association. 2015;77:34–43. Epub 2015/01/13. 10.1016/j.fct.2014.12.013 ; PubMed Central PMCID: PMCPmc4522946. 25572524 PMC4522946

[28] Muhlethaler-Mottet A, Balmas K, Auderset K, Joseph JM, Gross N. Restoration of TRAIL-induced apoptosis in a caspase-8-deficient neuroblastoma cell line by stable re-expression of caspase-8. Annals of the New York Academy of Sciences. 2003;1010:195–9. . 15033719 10.1196/annals.1299.033

[29] Muhlethaler-Mottet A, Bourloud KB, Auderset K, Joseph JM, Gross N. Drug-mediated sensitization to TRAIL-induced apoptosis in caspase-8-complemented neuroblastoma cells proceeds via activation of intrinsic and extrinsic pathways and caspase-dependent cleavage of XIAP, Bcl-xL and RIP. Oncogene. 2004;23(32):5415–25. 10.1038/sj.onc.1207704 . 15094781

[30] Muhlethaler-Mottet A, Flahaut M, Bourloud KB, Auderset K, Meier R, Joseph JM, et al Histone deacetylase inhibitors strongly sensitise neuroblastoma cells to TRAIL-induced apoptosis by a caspases-dependent increase of the pro- to anti-apoptotic proteins ratio. BMC cancer. 2006;6:214 10.1186/1471-2407-6-214 16930472 PMC1569857

[31] Chaudhary PM, Eby M, Jasmin A, Bookwalter A, Murray J, Hood L. Death receptor 5, a new member of the TNFR family, and DR4 induce FADD-dependent apoptosis and activate the NF-κB pathway. Immunity. 1997;7(6):821–30. 9430227 10.1016/s1074-7613(00)80400-8

[32] Peuhu E, Paul P, Remes M, Holmbom T, Eklund P, Sjoholm R, et al The antitumor lignan Nortrachelogenin sensitizes prostate cancer cells to TRAIL-induced cell death by inhibition of the Akt pathway and growth factor signaling. Biochemical pharmacology. 2013;86(5):571–83. Epub 2013/06/12. 10.1016/j.bcp.2013.05.026 . 23747345

[33] Peuhu E, Rivero-Muller A, Stykki H, Torvaldson E, Holmbom T, Eklund P, et al Inhibition of Akt signaling by the lignan matairesinol sensitizes prostate cancer cells to TRAIL-induced apoptosis. Oncogene. 2010;29(6):898–908. Epub 2009/11/26. 10.1038/onc.2009.386 . 19935713

[34] Cheng G, Zielonka J, McAllister D, Tsai S, Dwinell MB, Kalyanaraman B. Profiling and targeting of cellular bioenergetics: inhibition of pancreatic cancer cell proliferation. British journal of cancer. 2014;111(1):85–93. 10.1038/bjc.2014.272 24867695 PMC4090735

[35] Chang HH, Lee H, Hu MK, Tsao PN, Juan HF, Huang MC, et al Notch1 expression predicts an unfavorable prognosis and serves as a therapeutic target of patients with neuroblastoma. Clinical cancer research: an official journal of the American Association for Cancer Research. 2010;16(17):4411–20. 10.1158/1078-0432.CCR-09-3360 . 20736329

[36] Ferrari-Toninelli G, Bonini SA, Uberti D, Buizza L, Bettinsoli P, Poliani PL, et al Targeting Notch pathway induces growth inhibition and differentiation of neuroblastoma cells. Neuro-oncology. 2010;12(12):1231–43. 10.1093/neuonc/noq101 20716592 PMC3018939

[37] King D, Yeomanson D, Bryant HE. PI3King the lock: targeting the PI3K/Akt/mTOR pathway as a novel therapeutic strategy in neuroblastoma. Journal of pediatric hematology/oncology. 2015;37(4):245–51. Epub 2015/03/27. 10.1097/MPH.0000000000000329 . 25811750

[38] Mei H, Wang Y, Lin Z, Tong Q. The mTOR signaling pathway in pediatric neuroblastoma. Pediatr Hematol Oncol. 2013;30(7):605–15. Epub 2013/05/24. 10.3109/08880018.2013.798058 . 23697980

[39] de Oliveira MR, Ferreira GC, Schuck PF, Dal Bosco SM. Role for the PI3K/Akt/Nrf2 signaling pathway in the protective effects of carnosic acid against methylglyoxal-induced neurotoxicity in SH-SY5Y neuroblastoma cells. Chemico-biological interactions. 2015;242:396–406. 10.1016/j.cbi.2015.11.003 . 26577515

[40] Bahmad HF, Mouhieddine TH, Chalhoub RM, Assi S, Araji T, Chamaa F, et al The Akt/mTOR pathway in cancer stem/progenitor cells is a potential therapeutic target for glioblastoma and neuroblastoma. Oncotarget. 2018;9(71):33549–61. Epub 2018/10/17. 10.18632/oncotarget.26088 30323898 PMC6173359

[41] Kang Y, Park MA, Heo SW, Park SY, Kang KW, Park PH, et al The radio-sensitizing effect of xanthohumol is mediated by STAT3 and EGFR suppression in doxorubicin-resistant MCF-7 human breast cancer cells. Biochimica et biophysica acta. 2013;1830(3):2638–48. 10.1016/j.bbagen.2012.12.005 . 23246576

[42] Vanhoecke BW, Delporte F, Van Braeckel E, Heyerick A, Depypere HT, Nuytinck M, et al A safety study of oral tangeretin and xanthohumol administration to laboratory mice. In vivo. 2005;19(1):103–7. . 15796161

[43] Dorn C, Bataille F, Gaebele E, Heilmann J, Hellerbrand C. Xanthohumol feeding does not impair organ function and homoeostasis in mice. Food and chemical toxicology: an international journal published for the British Industrial Biological Research Association. 2010;48(7):1890–7. 10.1016/j.fct.2010.04.030 . 20427021

[44] Van Breemen RB, Yuan Y, Banuvar S, Shulman LP, Qiu X, Alvarenga RFR, et al Pharmacokinetics of prenylated hop phenols in women following oral administration of a standardized extract of hops. Molecular nutrition & food research. 2014;58(10):1962–9. 25045111 10.1002/mnfr.201400245 PMC4265473

[45] Fulda S, Kufer MU, Meyer E, van Valen F, Dockhorn-Dworniczak B, Debatin KM. Sensitization for death receptor- or drug-induced apoptosis by re-expression of caspase-8 through demethylation or gene transfer. Oncogene. 2001;20(41):5865–77. Epub 2001/10/11. 10.1038/sj.onc.1204750 . 11593392

[46] Hinz S, Trauzold A, Boenicke L, Sandberg C, Beckmann S, Bayer E, et al Bcl-XL protects pancreatic adenocarcinoma cells against CD95- and TRAIL-receptor-mediated apoptosis. Oncogene. 2000;19(48):5477–86. Epub 2000/12/15. 10.1038/sj.onc.1203936 . 11114725

[47] Eggert A, Grotzer MA, Zuzak TJ, Wiewrodt BR, Ho R, Ikegaki N, et al Resistance to tumor necrosis factor-related apoptosis-inducing ligand (TRAIL)-induced apoptosis in neuroblastoma cells correlates with a loss of caspase-8 expression. Cancer research. 2001;61(4):1314–9. Epub 2001/03/14. . 11245427

[48] Horak P, Pils D, Haller G, Pribill I, Roessler M, Tomek S, et al Contribution of epigenetic silencing of tumor necrosis factor-related apoptosis inducing ligand receptor 1 (DR4) to TRAIL resistance and ovarian cancer. Molecular cancer research: MCR. 2005;3(6):335–43. Epub 2005/06/24. 10.1158/1541-7786.MCR-04-0136 . 15972852

[49] Ozoren N, El-Deiry WS. Cell surface Death Receptor signaling in normal and cancer cells. Seminars in cancer biology. 2003;13(2):135–47. Epub 2003/03/26. . 12654257 10.1016/s1044-579x(02)00131-1

[50] Pennarun B, Meijer A, de Vries EG, Kleibeuker JH, Kruyt F, de Jong S. Playing the DISC: turning on TRAIL death receptor-mediated apoptosis in cancer. Biochimica et biophysica acta. 2010;1805(2):123–40. Epub 2009/12/08. 10.1016/j.bbcan.2009.11.004 . 19961901

[51] Bin L, Thorburn J, Thomas LR, Clark PE, Humphreys R, Thorburn A. Tumor-derived mutations in the TRAIL receptor DR5 inhibit TRAIL signaling through the DR4 receptor by competing for ligand binding. The Journal of biological chemistry. 2007;282(38):28189–94. Epub 2007/08/02. 10.1074/jbc.M704210200 . 17666396

